# Acceptability, Feasibility, and Outcome Responsiveness of the Joint Effort Mobile App for Promoting Lower-Risk Cannabis Use Among Young Adults: Pilot Randomized Controlled Trial

**DOI:** 10.2196/71957

**Published:** 2026-05-28

**Authors:** José Côté, Gabrielle Chicoine, Patricia Auger, Billy Vinette, Geneviève Rouleau, Marc-André Maheu-Cadotte, M Gabrielle Pagé, Judith Lapierre, Shalini Lal, Christine Genest, Guillaume Fontaine, Sylvie Cossette, Jinghui Cheng, Didier Jutras-Aswad

**Affiliations:** 1 Faculty of Nursing, Université de Montréal, 2375 Chem. de la Côte-Sainte-Catherine, Montreal, QC, H3S 2N4, Canada, 1 514-343-6437; 2 Research Centre, Centre Hospitalier de l’Université de Montréal, Montreal, QC, Canada; 3 Research Chair in Innovative Nursing Practices, Montreal, QC, Canada; 4 Knowledge Translation Program, Li Ka Shing Knowledge Institute, St. Michael's Hospital, Toronto, ON, Canada; 5 Department of Nursing, Université du Québec en Outaouais, Gatineau, QC, Canada; 6 Institute for Health System Solutions and Virtual Care, Women's College Hospital, Toronto, ON, Canada; 7 Institut du Savoir Montfort, Ottawa, ON, Canada; 8 Centre de recherche du Centre intégré de santé et de services sociaux de l’Outaouais, Gatineau, QC, Canada; 9 Department of Anesthesiology and Pain Medicine, Faculty of Medecine, Université de Montréal, Montreal, QC, Canada; 10 Faculty of Nursing, Université Laval, Québec, QC, Canada; 11 Center for Research on Social Innovations, Université Laval, Québec, QC, Canada; 12 School of Rehabilitation, Faculty of Medicine, Université de Montréal, Montreal, QC, Canada; 13 Research Centre, Douglas Mental Health University Institute, Montreal, QC, Canada; 14 Centre d’étude sur le trauma, Centre de recherche de l’Institut universitaire de santé mentale de Montréal, Montreal, QC, Canada; 15 Ingram School of Nursing, Faculty of Medicine and Health Sciences, McGill University, Montreal, QC, Canada; 16 Centre for Clinical Epidemiology, Lady Davis Institute for Medical Research, Jewish General Hospital, Montreal, QC, Canada; 17 Centre for Nursing Research, Jewish General Hospital, Montreal, QC, Canada; 18 Viral Hepatitis Clinical Research Program, Kirby Institute, University of New South Wales, Sydney, Sydney, Australia; 19 Centre for Implementation Research, Methodological and Implementation Research Program, Ottawa Hospital Research Institute, Ottawa, ON, Canada; 20 Montreal Heart Institute, Montreal, QC, Canada; 21 Department of Computer Engineering and Software Engineering, Polytechnique Montréal, Montreal, QC, Canada; 22 Department of Psychiatry and Addictology, Faculty of Medicine, Université de Montréal, Montreal, QC, Canada

**Keywords:** mobile health, mHealth, app, digital health applications, cannabis, behavior change, young adults, students, harm reduction

## Abstract

**Background:**

Cannabis use (CU) among young adults continues to be an important public health issue. Interventions to support lower-risk CU during young adulthood can improve health outcomes. Mobile applications constitute a promising mode of service delivery. However, there is a lack of evidence-based apps specifically developed for young adult cannabis users.

**Objective:**

This study aimed to evaluate the acceptability of a novel mobile app intervention (Joint Effort) and to assess the feasibility and outcome responsiveness of the study procedures used.

**Methods:**

A pilot study with a parallel-group randomized trial design was conducted with Canadian-based university students aged 18‐30 years reporting using cannabis ≥1 day in the past month. Participants were randomly assigned on a 1:1 ratio to either an experimental group (EG) involving the use of the Joint Effort mobile app or to a control group (CG) involving a web-based brief normative feedback message. The Joint Effort mobile app was designed to support CU self-management. This theory-informed behavior change intervention aims to reinforce the use of protective behavioral strategies by targeting intention, attitude, social norms, and self-efficacy. The app’s acceptability was assessed via uptake, engagement, and appreciation. The feasibility of study procedures was assessed via recruitment time, recruitment rate, and attrition rate. Outcome responsiveness was informed by participant-reported outcomes: CU frequency, intention to take action on CU, protective behavioral strategies use, severity of dependence, and psychological distress. All data were collected using a web-based survey at baseline, one-month (T1), and 2-month (T2) postbaseline. Descriptive analyses were carried out on all outcomes.

**Results:**

The recruitment period lasted 124 days, and the recruitment rate was 56% (99/178). The final dataset analyzed included 80 participants (39 in EG and 41 in CG). Mean age was 23.4 (SD 2.6) years, and 66% (53/80) self-identified as women. Study attrition was 18% (14/80). User uptake of the Joint Effort app (ie, proportion of participants in the EG who downloaded the app) was estimated at 59% (23/39), and the average time spent on it per participant was 8.2 minutes (SD 7.3; median 7.5, IQR 5.7). The app obtained a mean total score on the User Engagement Scale-Short Form of 3.8/5 (SD 0.5) and a mean app quality total score of 4.2/5 (SD 0.5) on the end user version of the Mobile App Rating Scale. The proportion of participants who reported daily CU in the past month decreased from 13% (5/39) at baseline to 4% (1/24) at T2 in the EG and from 7% (3/41) to 6% (2/36) in the CG.

**Conclusions:**

Joint Effort appears to be a promising, acceptable, and scalable mobile app to help young adult cannabis users who wish to better manage their CU. Findings should inform future randomized controlled trials to assess the efficacy of this mobile-based intervention for cannabis users.

## Introduction

### Background

Cannabis is one of the most commonly used substances for nonmedical purposes throughout the world. The prevalence of past-year cannabis use (CU) in the global community 15‐64 years of age in 2018 was estimated at 3.8%, which represented nearly 200 million people [1]. Attitudes toward CU have evolved substantially over the past 25 years [2]. The legalization of medical and recreational CU has expanded in Canada and the United States, and the legal status of CU has been shifting in other countries as well [3]. In the last decade, the consumption of cannabis products has increased considerably, particularly among young adults, typically 18‐25 years old [4-6]. In this population group, the prevalence of past-year CU was found to be 25% or higher in high-use regions such as North America, Oceania, and West Africa [1]. Moreover, and important for how it might impact potential life-course outcomes, the median age of onset of regular CU is 18‐19 years [7], and CU level and frequency begin to increase in very early adulthood and peak in middle age [8,9].

CU is associated with a wide range of health harms, including risk for CU disorder [10,11], short-term acute risks (eg, cannabis-impaired driving and related injuries/death, acute intoxication, impaired cognitive functioning), and other negative social consequences (eg, poor schooling or work performance, social isolation, family violence, stigmatization, poor living conditions) [12-14]. Importantly, CU has been linked to long-term adverse health outcomes such as cardiovascular problems, brain development issues, lung tissue injury, and mental health problems [15-18]. In the context of increased availability of high-THC products, country- or state-level legalization reforms allowing recreational CU, and public opinion to the effect that cannabis is a low-risk substance [6,19,20], there is a need for interventions to promote lower-risk CU and to prevent escalation of CU-related consequences among young adults.

Across the intervention continuum, CU interventions range from public awareness campaigns and prevention practices to treatment programs for CU disorder. Among these, lower-risk CU interventions [10] constitute a wide range of public health prevention or intervention tools inspired by harm reduction, a framework that places substance use on a spectrum from total abstinence to continued heavy use [21]. Generally, lower-risk CU interventions are based on health behavior change theories [22-24] and existing harm-reduction prevention approaches (eg, low-risk drinking guidelines, guidance for safe drug use practices) [25,26].

In recent years, particularly, there has been growing interest in the development of digital interventions for the promotion of lower-risk CU [27-30]. Digital modalities not only render interventions more accessible and cost-effective, but they also offer innovative opportunities for delivering personalized and real-time adaptive interventions that meet the needs and preferences of young adults [31-33]. An important body of literature documents the development, adaptation, and evaluation of digital interventions for lower-risk CU among young adults designed to be accessed online (ie, web- or internet-based) or via a mobile device (eg, cell phone, tablet, smartwatch) [28,29,34-40]. Altogether, the scientific evidence on the effects of digital interventions for lower-risk CU on health outcomes has been mixed. Specifically, while several studies have shown that digital interventions can reduce CU among young adults [36-42], other studies have found no effects on use [35] and cannabis-related consequences [34]. Furthermore, a scoping review by Sedrati et al [39] and a recent systematic review by Côté et al [34] both concluded that, despite considerable advances in digital interventions for lower-risk CU among young adults, most of the interventions developed and tested to date are web-based programs. These asynchronous digital tools typically use websites or online platforms, accessed mainly via computers, and consist primarily of didactic, static information (eg, facts, psychoeducational material) [40]. This means that content is neither individualized to the needs and characteristics of intended end users nor tailored in real time. Therefore, more research is needed to develop and test the acceptability and effectiveness of digital interventions using next-generation mobile health (mHealth) tools (eg, mobile apps, social media) that, undoubtedly, would be more consistent with the technological habits of today’s young adults.

Related to these concerns, digital CU interventions for young adults also pose unique challenges in terms of implementation and utilization. For one, adequate engagement is crucial for digital interventions to be successful [43,44]. However, research has shown that engagement tends to be low and that attrition is common [33,35]. This concern is particularly salient with respect to the subpopulation of young adults who do not perceive their CU as problematic and, consequently, have lower readiness to change. In addition, existing digital CU interventions were developed primarily for individuals with a CU disorder (5.1% of people aged 12 years or older in the United States) [45] or mental health needs [31,38,46]. This excludes a larger proportion of young adults who do not experience severe problems from their CU [37]. For example, recent studies have demonstrated the benefits of digital interventions aimed at supporting young adults after substance-use treatment programs [47-49]. However, these interventions are not tailored to individuals with lower severity substance-use who are not necessarily seeking to change their behavior or to prevent relapse [33]. Finally, many digital tools developed specifically for young adult cannabis users include therapist support [50-56]. From a public health perspective, this reduces scalability and may be unnecessary for people with lower CU severity.

In 2018, recognizing the need for a publicly available evidence-based app to promote lower-risk CU tailored to the needs of young adults [57], we developed a mobile app named Joint Effort. The detailed rationale for developing this new intervention is described elsewhere [58]. This mobile app aims to support CU self-management and reinforce protective behavioral strategies (PBS) use among young adult cannabis users. The intervention applies behavior change principles [59,60] that have previously demonstrated their efficacy in trials of interventions targeting risky behaviors [21,61,62]. Consistent with the current best practice in developing mHealth apps [63,64], our team’s prior work used an iterative intervention mapping and co-design approach [65-67] in a series of formative studies [58,68,69] to develop, inform, and refine Joint Effort. Additional formative work was required to determine the app’s acceptability and feasibility before its efficacy and effectiveness in improving health outcomes among young adult cannabis users could be tested. Against this backdrop, we conducted a pilot parallel-arm randomized controlled trial (RCT) to assess the acceptability of the Joint Effort app in terms of user uptake, engagement, and appreciation in a sample of young adult university students. We also assessed the feasibility of our study procedures and the responsiveness of outcome measures.

### Aim and Objectives

The objectives of this study were threefold:

1. To assess the acceptability of the Joint Effort app in terms of user uptake, engagement, and appreciation.
2. To assess the feasibility of study procedures and document the research process throughout the study period.
3. To assess outcome responsiveness in participant-reported outcomes, including CU, intention to take action on CU, PBS use, severity of dependence, and psychological distress.

## Methods

### Study Design

The study design was a pilot RCT [70] where a sample of young adult university students who reported CU in the past month was split into two parallel arms: (1) an experimental group (EG) that was given access to the Joint Effort mobile app; and (2) a control group (CG) that received one brief normative web-based feedback message regarding their CU and had access to publicly available information on lower-risk CU guidelines.

The study was registered in the ClinicalTrials.gov Protocol Registration and Results System (NCT05099016). The study reporting followed the CONSORT extension for Pilot and Feasibility Trials [71] checklist (Checklist 1) in reporting the trial.

### Setting and Sample

The target population for this study consisted of young adults studying at the Université de Montréal, a world-class, predominantly French-language university with approximately 67,000 students, located in a metropolitan region of Eastern Canada. We used a recruitment strategy centered on social media ads and posters on university campuses to obtain a broad and diverse sample of university student cannabis users. Individuals were eligible for this study if they met four criteria: (1) 18 to 30 years of age; (2) enrolled in an undergraduate or graduate program at the Université de Montréal; (3) self-reported at least one episode of past-month CU; and (4) had an iPhone to download the app to (Joint Effort was available only as an iOS app at the time of study).

Teare et al [72] recommend that at least 70 participants (35 per group) are needed in a pilot RCT to ensure a statistically precise measurement of each variable. To compensate for potential attrition at follow-up (ie, we expected up to 35% attrition at the 2-month postbaseline assessment) [34,35], we sought to recruit a total of 110 participants in order to end up with a final sample size of at least 70 completers.

### Intervention Characteristics: A Mobile App for Lower-Risk CU

**Figure 1.**
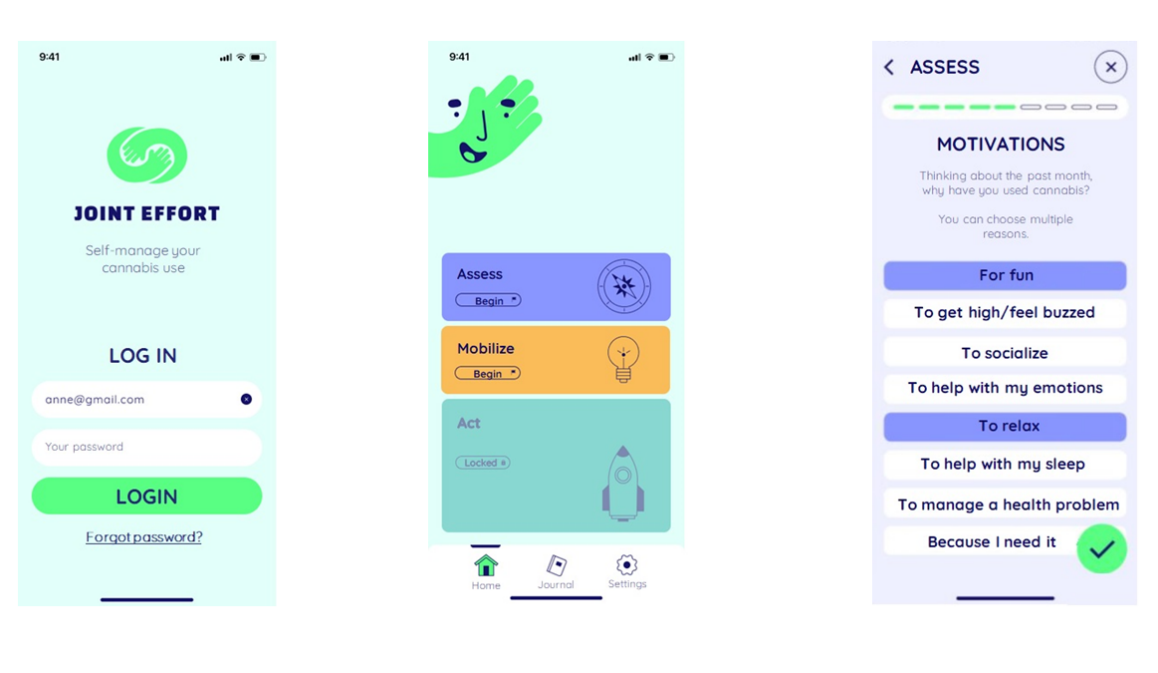
Screenshots from the Joint Effort mobile app.

The Joint Effort mobile app was designed to promote CU self-management and reinforce PBS use among young adult cannabis users. Developed based on Ajzen’s Theory of Planned Behavior [59], Joint Effort focuses on 4 core determinants, namely, intention, attitude, social norms, and self-efficacy. To address these, various evidence-based, theory-driven intervention strategies and methods are used, including personalized feedback, persuasive communication, positive reinforcement, self-observation, and activation of intention. A detailed account of how the principles of the Theory of Planned Behavior were integrated in the app is presented elsewhere [58]. The intervention content is organized into five sections: (1) “Assess” to allow users to gain a higher awareness of their CU, (2) “Mobilize” to support their decision-making process, (3) “Act” to guide them in establishing an action plan, (4) “Strengthen” to consolidate their change in behavior, and (5) “Observe” to monitor their CU. It addresses various topics such as CU prevalence, potential consequences, PBS, benefits of change, and possible difficult situations. The information is provided through general information messages, questionnaires (checkbox answers, reflection questions), and personalized feedback. The app also includes an optional consumption diary where users can enter information regarding their CU and customize certain features of their logbook for personalized monitoring (eg, frequency of daily entries, format, and concentration of cannabis used, personal notes). The intervention was designed as a self-managed exercise in prevention, given that it proposes simple strategies easy to apply and adopt without the help of outside resources. Joint Effort was available only in French and as an iOS mobile app (ie, for iPhones running on iOS version 13 or 14) at the time of the study. A detailed description of the mobile app intervention and its content, in accordance with the Template for Intervention Description and Replication (TIDieR) [73] Checklist, is available in Checklist 2 and in a publication elsewhere [58]. Figure 1 presents screenshots from the Joint Effort mobile app showing its general look and feel, as well as how features are organized within the app.

### Study Groups

Participants randomly assigned to the EG were invited to download and use the Joint Effort mobile app on their iPhones. Following randomization, members of the research team created a user account for each of them based on their chosen username/email collected during study registration. Participants received an automatically personalized email containing an App Store link to download the mobile app. Access to Joint Effort was unlimited in terms of frequency and duration of use over 8 weeks once their profile had been created by the research team. To optimize engagement with the intervention, optional pop-up notifications were embedded within the mobile app. Two types of notifications were sent: (1) to encourage users to continue with their action plan (scheduled 3, 5, and 7 days after completion of the “Act” section) and (2) to remind users about the app (triggered 9 days after their last login).

Participants randomly assigned to the CG received one brief normative web-based feedback message regarding their CU based on their past-month frequency of use reported during the baseline assessment. They were also sent links to access standardized information about lower-risk CU guidelines on reliable French public websites tailored for the Quebec context (eg, public health agencies, Quebec ministries of health). A single reminder inviting them to visit (or revisit) the recommended websites was sent via email 7 days after baseline.

### Randomization and Blinding

As recommended in the CONSORT guidelines [71,74], participants were randomized only after having provided consent and completed a web-based survey at baseline. Participants had an equal chance of being allocated to the EG or the CG (1:1 ratio). The allocation sequence was generated through a permuted block randomization list to ensure that the 2 groups were as even as possible during data collection. The allocation process (concealment and implementation) was entirely computerized. The sequence was programmed within the study website, and participants were automatically informed of the intervention they were assigned to by email. The study was partially single-blinded in that participants in the 2 study groups knew the intervention they were working with, but were unaware whether it was the experimental condition or not. The research coordinators were not blinded to assignments since they had to create unique profiles for each participant and to monitor follow-up.

### Measures

Acceptability was defined as the “suitability of the intervention from the perspective of the population of interest” [75], that is, young adult cannabis users. Acceptability of the Joint Effort mobile app was assessed via 3 distinct yet interrelated constructs, namely, uptake, engagement, and appreciation, only among participants from the EG.

Uptake was defined as the act of downloading the Joint Effort mobile app via the Apple iOS App Store [76]. Uptake rate was measured as a percentage calculated by comparing the number of EG participants who downloaded Joint Effort against the total number of participants randomized to the EG.

Engagement was defined as a quality of the user experience [77] characterized by a substantial exposure to the intervention content among uptakers [78]. Engagement was assessed among participants randomized to the EG using a hybrid approach [79] that included both objective and subjective measures: (1) quantity of exposure, that is, frequency and duration of use of Joint Effort, was collected automatically when users logged in to the mobile app (data extracted by the research team at the end of the 2-month intervention period); and (2) self-report measure of user engagement using the User Engagement Scale-Short Form (UES-SF) [80] after one month of using the app to minimize memory bias. Used in various digital domains, the UES-SF is a self-completed questionnaire that has demonstrated adequate construct validity and good internal consistency [80-82]. It consists of 12 items meant to measure 4 dimensions of engagement across 4 subscales labeled Aesthetic appeal, Focused attention, Perceived usability, and Reward factor (possible total score range: 12‐60). In this study, a French adaptation of the original English version of the UES-SF was used [83]. This French translation has shown satisfying preliminary psychometric properties in a sample of 57 Canadian-based university students (McDonalds’ omega [*ω*] coefficients of the French UES-SF subscales range from 0.8 to 0.9) [83].

Participant appreciation of Joint Effort was assessed with the end user version of the Mobile App Rating Scale (uMARS) [84]. Considering the detailed nature of the uMARS items, this questionnaire was completed 2 weeks after randomization to minimize memory bias. The uMARS is a reliable method to assess the quality of mobile-app tools that has demonstrated excellent internal consistency with a Cronbach α of 0.90 for the full scale, high internal consistency for the subscales, and good levels of interclass correlation coefficients at follow-up [84]. The uMARS comprises 20 items across 4 objective quality subscales—Engagement, Functionality, Aesthetic, and Information—and a subjective quality subscale, all rated on a 5-point Likert scale (possible total score range: 20‐100). Stoyanov et al [84] suggested determining the app quality mean score by computing the mean score for each objective quality subscale and averaging the four. The uMARS was translated into French by our research team following a rigorous translation process, similar to the one described in Côté et al [68,69].

Feasibility was defined as success in executing the study design and research procedures as planned [75]. In this study, it was assessed based on 3 parameters: recruitment feasibility, study attrition, and research procedures.

Recruitment feasibility was assessed by measuring recruitment time and recruitment rate. The recruitment time was calculated as the number of days from the time the study was advertised to the time our target sample size of 110 randomized participants was reached. The recruitment rate was measured as a percentage calculated by comparing the number of participants randomized to the study groups against the total number of individuals deemed eligible to participate [85].

The study attrition rate was measured as a percentage calculated by comparing the number of participants enrolled in the study who completed only the baseline assessment (ie, noncompleters) against the number of those enrolled in the study who completed the baseline assessment and at least one follow-up survey (ie, completers).

We also sought to determine the feasibility of the research procedures of a study conducted entirely via the web. To this end, we documented any issue that arose during recruitment or data collection (eg, data security and privacy, filtering fraudulent applications and information), as well as app-level problems (eg, technical issues with the operating system, challenges with software updates, and screen functionality).

The outcome responsiveness was assessed based on the following patient-reported outcomes: CU, intention to take action on CU, PBS use, severity of cannabis dependence, and psychological distress.

CU was measured by frequency of use via the following question adapted from the French version of the Canadian Community Health Survey [86]: “How often did you use cannabis in the past month?.” The extent to which participants experienced days of CU in the past month was assessed on the following 6-category rating scale: never; one day in the past month; 2-3 days in the past month; 1-2 days per week in the past month; 3-4 days per week in the past month; 5-6 days per week in the past month; and every day.

Intention to take action on CU referred to “readiness to engage in a behavior” [87] and was assessed via a 3-item French-language questionnaire developed for the purpose of this study, based on Ajzen’s guidelines for measuring Theory of Planned Behavior variables [88]. The questionnaire consisted of the following three items: (1) “I intend to take action on my CU in the coming month”; (2) “In the coming month, the chances that I will take action on my CU are…”; and (3) “In the coming month, I will take action on my cannabis use.” These were rated on a 7-point Likert scale ranging, respectively, from “strongly disagree” to “strongly agree,” “very low” to “very high,” and “very unlikely” to “very likely.” The final measure of intention to change was arrived at by summing the numbers from 1 to 7 corresponding to answers on the 3 items (possible score range: 3 to 21). The lower the total score, the weaker the intention to take action on CU in the coming month. In a previous study among young cannabis users, this scale showed good internal consistency with a Cronbach α of 0.87 [89].

PBS use was assessed via the short form of the 17-item Protective Behavioral Strategies for Marijuana (PBSM-17) Scale [60], a reliable instrument to identify and measure lower-risk CU strategies used by individuals before, during, and after CU. Methodological work was previously undertaken by our research team to culturally adapt and translate the PBSM-17 into French and evaluate its psychometric properties, as well as those of the original English version, in a sample of 211 bilingual, Canadian-based young adult university students who reported past-month CU [68,69]. Both the French and English versions demonstrated satisfactory psychometric properties, with good internal consistency (Cronbach *α*=0.88 and 0.91, for the French and English versions, respectively). Each item of the PBSM-17 concerns a particular PBS. Respondents are invited to rate the frequency of use for each strategy on a 6-point Likert scale ranging from never (value of 1) to always (value of 6). The PBSM-17 is scored by summing the values of the responses (possible score range: 17 to 102). The lower the total score, the lesser the use of PBSs.

Severity of cannabis dependence was assessed via the Severity of Dependence Scale (SDS) [90], a self-report instrument designed to measure the degree of dependence experienced by users of different types of drugs, including cannabis. A French language version of the SDS was used in this study [91]. The instrument consists of 5 items covering different psychological aspects of dependence: feeling of impaired control, anxiety, preoccupations, wish to stop, and compulsive use. The response choices for items 1 to 4 are scored as follows: 0=never/almost never, 1=sometimes, 2=often, and 3=always/nearly always. Those for item 5 are scored as follows: 0=not difficult, 1=quite difficult, 2=very difficult, and 3=impossible. The possible score ranges from 0 to 15. The cannabis version of the SDS has demonstrated satisfactory psychometric properties in samples of adults and adolescents from the general population as well as in clinical samples (eg, patients with a schizophrenia spectrum disorder) [92-96] and, more recently, it proved to have good internal consistency (Cronbach *α*=0.70) in a sample of 577 young adult (18‐30 y old) frequent and heavy cannabis users [97]. Previous studies have set empirically derived optimal cut-off scores for cannabis dependence at 2 to 4 or greater [92,96,98]. In this study, the cut-off score was set at 4 or greater.

Psychological distress was assessed via the Kessler Psychological Distress Scale–6 items (K6) [99], a psychometrically robust measure of psychological distress in adult populations used in epidemiological surveys globally, including the World Health Organization World Mental Health Survey [100]. The internal consistency of the K6 has been found to be adequate in general population samples (Cronbach *α*=0.8 to 0.9) [101-104], and one study [105] found the K6 to be valid and reliable in an epidemiological sample of Canadian youth (2010 respondents 15 to 19 years old), demonstrating a high internal consistency (Cronbach *α*=0.9). The K6 is a nonspecific measure of psychological distress consisting of 6 questions regarding whether the respondent had felt nervous, hopeless, restless, or fidgety in the past month, had felt so depressed that nothing could cheer them up, had felt that everything was an effort, and had felt worthless. Each item is rated on a 5-point Likert scale ranging from “none of the time” (value of 0) to “all of the time” (value of 4) (possible score range: 0‐24). A total score of 0 to 12 indicates that severe mental illness is unlikely; a total score of 13 or greater indicates that severe mental illness is likely [106]. The French translation of the scale was used [107].

### Study Procedures

The study was conducted entirely via web over a 6-month period (November 19, 2021, to May 20, 2022). It was hosted on a secure website developed for the purposes of this study. Recruitment was carried out via social media ads (ie, public and private Université de Montréal student Facebook groups) and posters on university campuses. Interested participants were directed to the study website to find out what the study was about. Once there, they were then asked to complete a web-based screening assessment (ie, 4 questions to confirm eligibility) and a consent form. Eligible participants were invited to register by choosing a username and providing contact information (ie, email address). To complete the registration process, participants had to click on a verification link sent by email, which allowed them to activate their profile and, subsequently, receive a unique link generated automatically to be directed to the web-based baseline survey to be completed.

The web-based baseline survey included a brief sociodemographic questionnaire specifically developed for the purposes of this study. It covered various individual-level parameters, including gender, age, ethnicity, educational level, and use of cannabis, alcohol, or other drugs (eg, cocaine, amphetamines, hallucinogens). The survey also included a question about CU frequency in the past month and instruments to assess outcome responsiveness (ie, intention to take action on CU, PBSM-17, SDS, and K6).

The same web-based survey without the sociodemographic questionnaire was administered to all participants at 2 other time points: 1 month (T1) and 2 months (T2) postbaseline. EG participants, who were invited to download the Joint Effort app and use it for 2 months, were also asked to complete questionnaires regarding acceptability variables, including the uMARS at 2 weeks postbaseline and the UES-SF scales at T1. A question asking participants to confirm their date of birth was included in the survey at each time point for verification purposes. Analytics data on participant use of Joint Effort, including quantity of exposure and usage patterns, were collected automatically by a web-based platform developed specifically for this study, hosted on a secure server. A research assistant could be contacted during the study period by email to answer questions and help participants with issues regarding the app and web-based surveys.

All data collected during the study period were stored digitally at the study website until downloaded onto a secure virtual server at the research center for analysis. Given that fraudulent (or inconsistent) data are a well-known problem in web-based survey studies [108,109], an in-depth examination of the data collected was carried out to assess their reliability and accuracy, and to double-check the identity of the participants before performing any statistical analysis. This involved verifying that the dates of birth provided by participants were consistent across time points and confirming that the email addresses provided by participants were valid. Participants with inconsistent data or contact information were considered “fraudulent participants” [110] and, therefore, not included in the final dataset analyzed.

### Data Analysis

All statistical analyses were conducted in R (version 4.2.3; R Foundation for Statistical Computing). Analyses were carried out by an independent, experienced biostatistician not blinded to participant group assignment. As per protocol, the biostatistician cleaned the dataset to enhance data consistency and quality. No interim analysis was performed, and, as recommended for pilot study designs [111], no hypothesis testing (ie, inferential analysis) was carried out. Descriptive analyses of sample characteristics included computing means and SDs for continuous variables and the frequency of distribution for categorical variables. Descriptive analyses were carried out on the acceptability and feasibility variables as well. Finally, descriptive statistics were calculated to explore outcome responsiveness, and the same analytical approach was used for all outcomes.

Descriptive analyses, including means and SDs for continuous variables and frequency of distribution for categorical variables, were conducted for sample characteristics, acceptability/feasibility variables, and outcome responsiveness.

### Ethical Considerations

Ethical approval was obtained from the Research Ethics Committee of the Centre Hospitalier de l’Université de Montréal (CHUM; #21.196) and from the Comité d’éthique de la recherche en sciences et en santé of the Université de Montréal (CERSES; #2021‐1223). Informed consent was obtained from all participants prior to study activities. Participants received financial compensation for their time and effort in the form of C $15 and C $25 (US $11 and US $18) Amazon.ca gift certificates at T1 and T2, respectively. All collected data have been deidentified, and results are reported at the sample level.

## Results

### Feasibility of Study Procedures

#### Recruitment Feasibility

**Figure 2.**
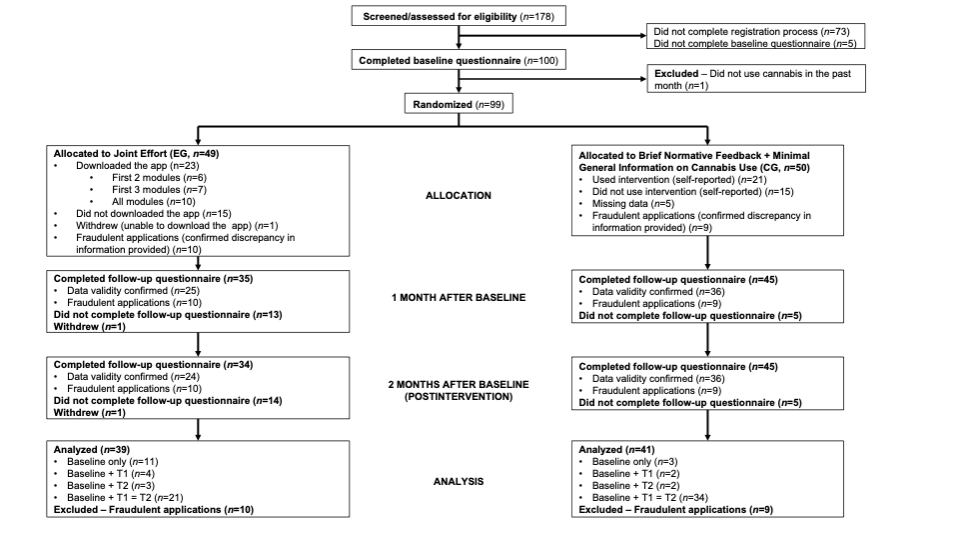
Study flow diagram with a pilot randomized controlled trial design based on Eldridge et al [70]. CG: control group; EG: experimental group.

As a result of our recruitment strategies, 178 people were deemed eligible after completing the screening survey (Figure 2 shows the study flow diagram). Of these, 73 were excluded for not completing the registration process, and 5 others for not completing the baseline survey. A total of 100 participants completed the baseline survey, and one participant was excluded afterward (ie, this participant was eligible according to the screening survey, but reported no CU in the baseline survey). The remaining 99 were randomized to the EG and CG, yielding a 56% (99/178) recruitment rate. Of these, 19 were considered fraudulent participants and eliminated for providing discrepant dates of birth or contact information on the registration form and the baseline or follow-up survey (10/49 participants in the EG and 9/50 participants in the CG). Consequently, there were 80 participants (39 EG and 41 CG) in the final dataset analyzed.

As for recruitment time, it took 124 days total from the day the study began to be advertised on social media and on campus to the day our target sample size of 110 randomized participants was reached (from November 19, 2021, to March 22, 2022).

#### Study Attrition

Of the 80 participants, 61 (76%) completed the assessment at one month postbaseline (T1) and 60 (75%) completed the assessment at 2 months postbaseline (T2). In all, 66 participants were considered completers for having completed the assessments at T0 and T1, T0 and T2, or all 3 assessments (ie, T0, T1, and T2). In the CG, 38 of 41 participants (93%) were considered completers, compared with 28 of 39 (72%) in the EG. The overall attrition rate was 18%, as 14 of the 80 participants (CG: 3/41; EG: 11/39) were considered noncompleters.

#### Sample Characteristics

The characteristics of participants at baseline are presented in Table 1. Age ranged from 19 to 29 years (mean 23.4, SD 2.6). Two-thirds identified as women (53/80, 66%), nearly 3-quarters were born in Canada (58/80, 73%), and 3-quarters were undergraduate students (60/80, 75%). Just under half were classified as occasional cannabis users (34/80, 43% CU 1-3 d/mo) and one out of ten as daily cannabis users (8/80, 10%). More than half of the participants reported using alcohol regularly (44/80, 55% alcohol use 1-4 d/wk), and the vast majority did not use other drugs (67/80, 84%).

**Table 1.** Participant characteristics at baseline (T0) for the overall sample (n=80), by study group and by study completion pattern.

Characteristic	Total sample (n=80)	Study groups		Study completion pattern	
		EG^a^ (n=39)	CG^b^ (n=41)	Completers (n=66)	Noncompleters (n=14)
Age (years), mean (SD)	23.4 (2.6)	23.6 (2.5)	23.3 (2.7)	23.5 (2.6)	22.7 (2.4)
Gender, n (%)					
Male	26 (33)	9 (23)	17 (41)	22 (33)	4 (29)
Female	53 (66)	29 (74)	24 (59)	43 (65)	10 (71)
Did not want to answer	1 (1)	1 (3)	0 (0)	1 (2)	0 (0)
Born in Canada, n (%)					
Yes	58 (73)	28 (72)	30 (73)	48 (73)	10 (71)
No	20 (25)	10 (26)	10 (24)	16 (24)	4 (29)
Did not want to answer	2 (3)	1 (3)	1 (2)	2 (3)	0 (0)
University program, n (%)					
Undergraduate	60 (75)	28 (72)	32 (78)	47 (71)	13 (93)
Master’s	12 (15)	6 (15)	6 (15)	12 (18)	0 (0)
Doctorate	7 (9)	5 (13)	2 (5)	6 (9)	1 (7)
Did not want to answer	1 (1)	0 (0)	1 (2)	1 (2)	0 (0)
Recruitment strategy^c^, n (%)					
Facebook ad	60 (75)	30 (77)	30 (73)	51 (72)	9 (64)
Posters on campus	8 (10)	4 (10)	4 (10)	6 (9)	2 (14)
Other^d^	15 (19)	6 (16)	9 (19)	12 (17)	3 (21)
Did not want to answer	2 (3)	1 (3)	1 (2)	2 (3)	0 (0)
Past-month cannabis use^e^, n (%)					
Occasional^f^	34 (43)	17 (44)	17 (41)	28 (42)	6 (43)
Regular^g^	38 (48)	17 (44)	21 (51)	32 (49)	6 (43)
Every day	8 (10)	5 (13)	3 (7)	6 (9)	2 (14)
Past-month alcohol use^e^, n (%)					
Never	12 (15)	7 (18)	5 (12)	10 (15)	2 (14)
Occasional^f^	23 (29)	10 (26)	13 (32)	19 (29)	4 (29)
Regular^g^	44 (55)	21 (54)	23 (56)	36 (55)	8 (57)
Every day	1 (1)	1 (3)	0 (0)	1 (2)	0 (0)
Past-month drug use^e^^h^, n (%)					
Never	67 (84)	33 (85)	34 (83)	57 (86)	10 (71)
Occasional^f^	13 (16)	6 (15)	7 (17)	9 (14)	4 (29)
Regular^g^	0 (0)	0 (0)	0 (0)	0 (0)	0 (0)
Every day	0 (0)	0 (0)	0 (0)	0 (0)	0 (0)

a EG: experimental group.

b CG: control group.

c More than one recruitment strategy possible.

d Other included: receiving a personalized email message or a friend referral.

e Frequency of use was determined by the number of days on which cannabis, alcohol, or other drugs were used in the past month.

f Occasional: 1‐3 days per month.

g Regular: 1‐6 days per week.

h Included but was not limited to cocaine, amphetamines, and hallucinogens.

### Joint Effort Acceptability

#### Uptake

Of the 39 participants randomized to the EG, 23 downloaded the Joint Effort mobile app to their iPhone for an uptake rate of 59%.

#### Engagement

Of the confirmed uptakers, 43% (10/23) viewed all the sections of the mobile app, while 26% (6/23) viewed only the first two (ie, “Assess” and “Mobilize”), and 30% (7/23) viewed only the first three (ie, “Assess,” “Mobilize,” and “Act”). In terms of quantity of exposure, the mean number of screens viewed per participant was 32.4 (Min-Max=15-89; SD 18.0). Regarding duration of exposure, participants spent an average of 8.2 minutes (Min-Max=53 s-33.5 min; SD 7.3 min) and a median of 7.5 minutes (IQR 5.7) using the app. The total period of use ranged from 1 to 59 days (mean 19.5; SD 21.9) with a 5-day median (IQR 36).

Regarding subjective engagement, the mean total score on the UES-SF was 3.8 (SD 0.46) at T1. Descriptive statistics for the UES-SF subscales and items are given in Table 2. Among the 4 UES-SF subscales, Aesthetic Appeal obtained the highest score (mean 4.3; SD 0.7), followed by Perceived usability (mean 4.2; SD 0.9), while Focused attention obtained the lowest score (mean 2.8; SD 0.9).

**Table 2.** Participant subjective engagement scores on the User Engagement Scale-Short Form (UES-SF), one-month postbaseline (T1) (n=18).

Subscale and item	Mean (SD)^a^
Focused attention	2.8 (0.9)
I lost myself in this experience.	2.2 (1.1)
The time I spent using Joint Effort just slipped away.	3.2 (0.9)
I was absorbed in this experience.	2.9 (0.9)
Perceived usability	4.2 (0.9)^b^
I felt frustrated while using this Joint Effort mobile app.	4.0 (1.1)^b^
I found this Joint Effort mobile app confusing to use.	4.1 (0.9)^b^
Using this Joint Effort mobile app was taxing.	4.4 (0.8)^b^
Aesthetic Appeal	4.3 (0.7)
This Joint Effort mobile app was attractive.	4.1 (0.9)
This Joint Effort mobile app was esthetically appealing.	4.4 (0.6)
This Joint Effort mobile app appealed to my senses.	4.3 (0.8)
Reward factor	4.0 (0.7)
Using Joint Effort mobile app was worthwhile.	4.1 (0.8)
My experience was rewarding.	3.6 (0.8)
I felt interested in this experience.	4.3 (0.6)
Overall score	3.8 (0.5)

a Possible score range of 1 to 5 (1=strongly disagree; 5=strongly agree); the higher the score, the higher the subjective engagement.

b Reverse scored. (1=strongly agree; 5=strongly disagree); the higher the score, the higher the level of perceived usability.

#### Appreciation of Joint Effort

Sixteen participants in the EG provided data regarding their appreciation of Joint Effort 2 weeks after randomization. The app obtained a mean app quality total score of 4.2 out of 5 (SD 0.5). Among the 5 uMARS subscales, Aesthetics (mean 4.4, SD 0.6), Information (mean 4.3, SD 0.5), and Functionality (mean 4.3, SD 0.5) scored high, while Engagement (mean 3.8, SD 0.6) and App subjective quality (mean 3.3, SD 0.6) scored lower (Multimedia Appendix 1). The Quantity of information item (”Is the information within the app comprehensive but concise?”) included in the Information subscale was the item that scored highest (mean 4.6, SD 0.6) on the 5-point Likert scale, while the Willingness to pay item (“Would you pay for this app?”) part of the App subjective quality scale scored lowest (mean 1.9, SD 0.8*).*

#### Outcome Responsiveness

Results for CU frequency, intention to take action on CU, PBS use, severity of cannabis dependence, and psychological distress among participants in the EG and the CG at the 3 measurement time points are presented in Table 3.

**Table 3.** Participant outcomes at baseline (T0) and follow-up assessments (T1, T2), by study group (n=80).

Variable	Baseline (T0)		1 month (T1)		2 months (T2)	
	CG^a^ (n=41)	EG^b^ (n=39)	CG (n=36)	EG (n=25)	CG (n=36)	EG (n=24)
Cannabis use (past month), n (%)						
Never	0 (0)	0 (0)	4 (11)	3 (12)	6 (17)	4 (17)
Occasional (1‐3 days/month)	17 (41)	17 (44)	11 (31)	7 (28)	16 (44)	8 (33)
Regular (1‐6 days/week)	21 (51)	17 (44)	19 (53)	13 (52)	12 (33)	11 (46)
Everyday	3 (7)	5 (13)	2 (6)	2 (8)	2 (6)	1 (4)
Intention to take action^c^, mean (SD)	12.5 (4.3)	12.4 (5.2)	13.6 (5.4)	14.3 (4.6)	13.4 (6.0)	14.3 (5.3)
PBS-17^d^, mean (SD)	72.9 (12.5)	73.7 (15.1)	76.4 (12)	78.8 (15.8)	80.2 (12.3)	80.0 (13.0)
SDS^e^^f^, n (%)						
No dependence	26 (65)	21 (54)	N/A^g^	N/A^g^	24 (71)	15 (68)
Dependence^h^	14 (35)	18 (46)	N/A^g^	N/A^g^	10 (29)	7 (32)
Psychological distress^i^^j^, n (%)						
Low/moderate^k^	28 (68)	28 (76)	N/A^g^	N/A^g^	24 (69)	20 (83)
Severe^l^	13 (32)	9 (24)	N/A^g^	N/A^g^	11 (31)	4 (17)

a CG: control group.

b EG: experimental group.

c Intention to take action on cannabis use; possible score range 3‐21.

d PBSM-17: 17-item Protective Behavioral Strategies for Marijuana Scale; possible score range 17‐102 (summed score).

e SDS: Severity of Dependence Scale (for cannabis); possible score range 0‐15.

f 1 missing data at T0, 4 missing data at T2.

g Outcome not assessed at T1.

h Cannabis dependence=score≥4.

i Kessler Screening Scale for Psychological Distress (K6); possible scores range 0‐24.

j 2 missing data at T0, 1 missing data at T2.

k Low/moderate psychological distress=scores between 0 and 13.

l Severe psychological distress=scores between 14 and 24.

Most participants in both the EG and the CG self-assessed their past-month CU frequency as between occasional and regular at baseline, and these proportions remained mostly similar at T1 and T2. The proportion of participants who reported daily CU in the past month decreased from 13% (5/39) at baseline to 4% (1/24) at T2 in the EG and from 7% (3/41) to 6% (2/36) in the CG.

Participants in both the EG and the CG expressed a moderate level of intention to take action on their CU at baseline, and this score tended to increase at T1 and T2 for participants in the EG. Similarly, participants in the EG and CG showed a moderate level of PBS use at baseline, and this result was found to increase in both groups at follow-up time points. Regarding psychological distress, the proportion of participants in the EG who scored a severe level of psychological distress decreased from 24% (9/39) at baseline to 17% at T2 (4/24), while in the CG, the proportion remained quite similar from baseline (13/41, 32%) to T2 (11/36, 31%). Finally, 46% (18/39) and 35% (14/41) of the participants in the EG and the CG, respectively, scored from 4 to 15 on the SDS at baseline, which indicated cannabis dependence. At T2, the proportions decreased to 32% (7/24) and 29% (10/36), respectively.

## Discussion

### Principal Findings

This pilot RCT sought to evaluate the acceptability of the Joint Effort mobile app in a sample of Canadian-based university students who used cannabis in the past month, examine the feasibility of the research procedures, and provide data regarding outcome responsiveness.

### Acceptability of the Joint Effort Mobile App

Engaging adequately with a mobile app presupposes first downloading it and then using it to an extent corresponding to the optimal exposure threshold. This usage will be largely—but not solely—influenced by the user’s appreciation of the app. Therefore, we set out to assess user acceptability of the Joint Effort app via 3 distinct but interrelated parameters, namely, uptake, engagement, and appreciation.

Almost 60% of the participants in the EG downloaded the app (23/39). Thus, many participants who decided to participate in this study failed to use the proposed mobile app. One explanation could be related to potential technical issues with accessing the app and user account setup. Some users contacted the research team for assistance reinitializing their password to access the app. We can hypothesize that others faced the same problem but did not bother to contact us. Various factors can influence uptake, such as basic technological skills, app awareness, app availability and accessibility, social influences, curiosity, and user guidance [76]. In the context of this study, lack of user guidance might have had a negative/deteriorative impact on uptake. Further exploration of barriers and facilitators to uptake and strategies to optimize app download is needed.

Results suggest that Joint Effort was well received, deemed easy to use, and favorably rated by young adult cannabis users who used the proposed mobile app. For instance, the app obtained a good overall score on the uMARS (4.20/5, SD 0.5). Aesthetics was the dimension that obtained the highest score, followed by information, functionality, engagement, and subjective quality. These results are consistent with those obtained by Santesteban-Echarri et al [49], who reported a good uMARS overall score (mean 3.8, SD 0.5) in their pilot test of a mobile-based app to monitor CU among youth at high clinical risk for psychosis.

Engaging with a mobile app requires an autonomous process that depends on one’s commitment to the behavior to be changed and one’s self-management capabilities. In a systematic review of influences on uptake of and engagement with health and well-being smartphone apps across various fields (41 studies), Szinay et al [76] found that rapid disengagement was common and that uptake and engagement appeared to be influenced by a wide range of individual-level factors related to capability, opportunity, and motivation. In this study, in terms of quantity of exposure, 43% (10/23) of the downloaders viewed all sections, while 57% (13/23) completed the first 2 or 3 only. Regarding duration of exposure, our results showed a wide variation across participants in terms of frequency and time of use. This last finding might reflect the different purposes for which Joint Effort was being used.

Relatedly, in a systematic review of the concept of adherence to electronic health technology (34 studies), Sieverink et al [112] found that most of the time participants did not use technologies as suggested, whether in terms of features used, frequency of use, time of use, or place of use. These findings raise an important question, namely, whether all users need to experience all elements of a technology to achieve effects, especially considering that the intended engagement with an app may vary based on the utility objectives of the app and desired outcomes, as well as the user’s personal goals and substance use behaviors. Successful engagement may not be a function solely of the amount of exposure (ie, the more use, the better). This emphasizes the importance of measuring engagement in different but complementary ways.

In this study, in addition to objective engagement (ie, frequency and duration of app usage data metrics collected automatically), we also measured subjective engagement using a self-reported questionnaire. The results showed that Aesthetic Appeal was the UES-SF subscale that obtained the highest score, followed by the Reward factor. Currently, there is no standardized approach in the existing body of literature to assessing engagement in mHealth interventions, as engagement depends on the targeted behaviors and how they are conceptualized, operationalized, and measured [113-115]. For instance, a scoping review of mHealth intervention apps [113] found that 20% of the studies included did not measure user engagement and, of those that did quantifiably (37/54), only 8% (3/37) used an established scale to evaluate engagement outcomes. Consequently, it is difficult to compare our results regarding user engagement with the Joint Effort app against those of other mobile app interventions for CU. Enhancing participant engagement with mHealth apps is undoubtedly a priority for wellness and health care. It remains a significant challenge for many app developers, researchers, and clinical teams [78,113].

### Feasibility of Study Procedures

Recruitment primarily via social media ads resulted in the recruitment of our target sample over the span of 18 weeks. This underscores the feasibility of such methods but also highlights that recruitment costs and duration are key factors that must be considered from the outset to ensure successful recruitment. Although representativeness in a small pilot study cannot be determined, larger studies support the utility of web-based recruitment approaches [116,117]. In this pilot study, the recruitment rate was comparable to results from prior formative research on digital interventions targeting young adult cannabis users [33,118,119].

The overall study attrition rate was 18% (14/80), though there were more noncompleters in the EG than in the CG at the 2-month postbaseline assessment. In a similar pilot study intended to examine the feasibility and acceptability of a smartphone app intervention for adult cannabis users seeking to reduce or cease use [119], attrition rates of 16% and 32% were reported at postintervention (after 4 weeks of using the app) and at one-month follow-up, respectively. Similarly, in a scoping review (54 studies) of the components of mHealth intervention apps that encourage or hinder engagement, participant retention ranged from 14% to 100%, the average was 68% (SD 25%), and the median 79% [113]. Notably, only one-third of the studies included in this review (18/54) reported retention rates of 80% or higher. Against these numbers, the attrition rate (14/80, 18%) or retention rate (66/80, 83%) observed in this study can be considered acceptable.

Participant recruitment and retention are recognized as two of the most important challenges when conducting clinical trials [120]. These challenges are all the greater when research is conducted online. For example, a systematic review and meta-analysis (17 studies) by Meyerowitz-Katz et al [121] found that the “pooled estimate for dropout rates in trials of app-based interventions for chronic diseases was 43% over a variety of timelines”. In another review (62 studies), Amagai et al [122] reported that mHealth apps were hampered by substantial participant dropout or attrition and suggested that factors related to particular app elements (eg, feedback, reminders, in-app support from coaches and peers) and research strategies (eg, compensation and niche samples) should be considered to promote retention. Of note, participant engagement in mHealth apps and study retention (or attrition) are distinct constructs that should not be used interchangeably. Engagement in a given intervention can affect a study’s retention/attrition rate. Given the interconnected nature of these constructs, an integrated approach to analyzing recruitment, retention/attrition, and engagement outcomes should be used in evaluations of mHealth apps to broaden the evidence base but, more importantly, to determine how different strategies impact these three (ie, participant recruitment, retention, and engagement).

A second feasibility objective of this pilot trial was to document the research process throughout the study period in order to pinpoint and learn from the challenges and other aspects of the procedures applied before moving on to conduct a large-scale web-based RCT of the Joint Effort app. A major challenge faced during this study was the detection and management of fraudulent applications and the identification of information provided by potentially eligible or eligible participants. After reviewing completed surveys for indicators of fraudulent or suspicious data, 19% (19/99) of enrolled participants were found to have provided inconsistent information and, subsequently, were excluded from analysis. Similarly, other web-based trials or survey studies that monitored misrepresentation and fraud have consistently reported fraud rates of 20% to 35% [123-126]. Unfortunately, fraudulent behavior is a fairly widespread phenomenon in online research [127-129], which can have a significant impact on data integrity and quality, sample size and composition, and statistical precision [108,130,131].

Some researchers have outlined processes or methods, indicators, and logistic frameworks to address potential fraud [129,132,133]. In this pilot study, several strategies were rigorously incorporated to prevent, detect, and respond to fraudulent behavior by applicants/participants. These included automated procedures to prevent the same individual from registering more than once with the same email or username, as well as human monitoring (eg, checking for multiple registrations occurring almost simultaneously, identifying suspicious/fake email addresses, and unrealistic survey completion times). We also included combinations of survey questions repeated at each measurement time point (baseline, T1, and T2) designed to identify inconsistent responses in a participant’s identity information (eg, date of birth). This method has been identified as an effective strategy for accurately detecting inconsistencies and preventing data quality threats, including fraudulent entries from automated bots and duplicate submissions [108,134,135]. Finally, financial compensation was given only upon completion of the follow-up assessments, as it has been shown that financial incentives can motivate applicants to register more than once [133] and relatively immediate payments have been found to yield greater rates of fraudulent behavior by participants [127].

Although all the strategies implemented in this pilot study certainly contributed to preventing, detecting, and handling fraud, the fact remains that it may be impossible to completely eliminate it. In an online cross-sectional study (414 survey entries), Ballard and colleagues [123] found that, consistent with our observations, even when protocols were put in place to improve fraud detection, 28.7% of submitted web-based surveys were “fraudulent” and an additional 10.1% were cases of “potential fraud.” These authors also suggested that researchers should have a fraud detection algorithm in place prior to data collection and use a combination of indicators and methods to systematically review all online applications and verify participant identity and eligibility [123]. Based on the results of this study and our experience designing and implementing pilot RCTs using social media recruitment and web-based data collection, we encourage researchers conducting studies of mHealth apps to be vigilant about the potential for fraud, to keep detailed records of the verification process, and to report their study findings accordingly. Ultimately, there is a need for further reflection regarding the advantages and disadvantages associated with fraud prevention and detection methods, given that ethical tensions can emerge between preserving the integrity of research versus protecting the privacy and confidentiality of study participants.

### End User Profile and Outcome Responsiveness

In terms of participant characteristics, a higher proportion of women was reached compared with men. This adds to the growing body of literature indicating that CU treatment-related differences exist between men and women [10,136-138]. Although help-seeking behaviors to address CU problems are rare among both young adult men and young adult women [139,140], women are more likely to seek out anonymous substance use resources, such as web-based or mobile app interventions [33,141]. Women may also more readily recognize the need to change their CU in an attempt to better manage problems related to their CU. Lending support to this hypothesis, research has shown that women often endorse external reasons for change, such as experiencing cannabis-related negative consequences and social acceptability, whereas men seem to be motivated more by internal reasons, such as self-control and self-efficacy [142-145]. This means that women may be more willing to make behavioral changes and access strategies and motivational support to prevent or reduce the consequences/risks associated with CU. Given that cannabis-related negative consequences are disproportionally more common among young adult males [10,146], it would be worthwhile for future studies to ask young men directly about what they consider motivational or attractive to initiate change. In sum, our findings highlight the challenges of reaching target populations and developing mobile app CU interventions that take into account gender differences across a range of other clinical correlates (eg, readiness to change, sources of motivation to behavior change) that can affect young adults in their decision to take steps towards better CU management.

Regarding change in outcomes over time, our results showed that participants in both the EG and the CG indicated a moderate level of intention to take action on CU at baseline, and that the scores in this regard tended to increase at the follow-up assessments for participants in the EG. Similarly, participants in the EG and the CG indicated a moderate level of PBS use at baseline, and this level was found to increase in both groups over time. Regarding CU frequency, most participants in both groups self-assessed it as occasional to regular at baseline, with similar proportions at T1 and T2. Moreover, from baseline to postintervention, the proportion of participants in the EG who reported daily CU in the past month decreased by 9%, and those who reached the cut-off for cannabis dependence decreased by 12%. Overall, our results indicate that participants responded positively to Joint Effort and demonstrated some potential for behavior change as a result.

### Limitations

This study, focusing on user acceptability, study feasibility, and outcome responsiveness, presents some limitations.

One of the major limitations related to our Internet-based recruitment strategy—which is an inherent challenge in online intervention trials—is the discovery of fraudulent identities after participants were enrolled in the study. This is a critical issue as it directly altered the randomization process and, to some extent, may have impacted the integrity of the research. Although several precautions were taken throughout the study to prevent and detect fraudulent applications using both automated detection procedures and human monitoring, our fraud detection methods were not infallible. This limitation is partially due to the lack of in-person interactions between research team members and participants, which increases the risk that applicants are not who they claim to be. The participation incentives offered in the study are another factor that may have contributed to increased fraud. A multi-layered approach to fraud prevention and detection may be warranted in future studies of mHealth apps to help reduce the inclusion of fraudulent applications [127,129,134]. Despite the numerous risks associated with web-based recruitment, this method nevertheless has significant advantages over other traditional recruitment strategies. This pilot study thus affords valuable lessons for subsequent testing of Joint Effort and highlights the need for careful implementation of processes to help address potential fraud in future online trials and in web-based survey research in general.

We focused our means of recruitment mainly on social media and posters on campus in an attempt to reach a diverse population of university students. This strategy allowed us to recruit young adult cannabis users from a large university student population and achieve our sample size goal over a relatively short period of time. However, this recruitment method may have introduced a self-selection bias. We certainly recruited people interested in engaging with the mobile app and motivated to better manage their CU, which may not be representative of the target population. Also, the fact that the app was available on iOS only might have led to a different sociodemographic sample. The decision to develop the mobile application on iOS was based on financial limitations (ie, the cost associated with the development on both smartphone operating systems exceeded the budget). Also, based on a previous study conducted among a similar sample [68,69], more respondents used an iPhone to complete the survey compared with an Android (respectively 41% vs 19%) (data not published). This target population likely had a higher level of digital literacy than the broader population, a factor that potentially limits the transferability of our findings.

Another limitation is related to samples of young adult cannabis users that were relatively small at each time point, with the EG experiencing greater attrition than the CG. Consequently, and despite the generally positive findings supporting the potential of the Joint Effort app, further study is required with a larger sample to determine not only the efficacy of Joint Effort on CU, but also the mediators to use (eg, intention to use, PBS) and the consequences of CU (eg, cannabis dependence). These findings justify further testing of Joint Effort in a fully powered trial.

### Conclusions

Most existing interventions for young adult cannabis users wishing to better manage their use are limited by low uptake due to accessibility and stigma concerns. Advancements in technology have brought new ways in which a wide range of digital interventions can be developed and delivered. Joint Effort offers the advantage of convenient delivery via a smartphone app, which considerably minimizes these accessibility and stigma concerns. This pilot study provides support for Joint Effort’s acceptability within the target population as an mHealth intervention for lower-risk CU and suggests that the study design used is feasible. This warrants future testing in a fully powered trial (ClinicalTrials.gov NCT05620433).

## Supplementary material

10.2196/71957 Multimedia Appendix 1 Joint Effort mean scores on the user version of the Mobile App Rating Scale (uMARS).

10.2196/71957 Checklist 1 CONSORT 2010 extension for Pilot and Feasibility Trials Checklist.

10.2196/71957 Checklist 2 TIDier Checklist.

## Abbreviations

CG: control group
CU: cannabis use
EG: experimental group
K6: Kessler Psychological Distress Scale–6 items
mHealth: mobile health
PBS: protective behavioral strategies
PBSM-17: 17-item Protective Behavioral Strategies for Marijuana
RCT: randomized controlled trial
SDS: Severity of Dependence Scale
TIDieR: Template for Intervention Description and Replication
UES-SF: User Engagement Scale-Short Form
uMARS: user version of the Mobile App Rating Scale
