# Supplementary material for: Acceptability, Feasibility, and Outcome Responsiveness of the Joint Effort Mobile App for Promoting Lower-Risk Cannabis Use Among Young Adults: Pilot Randomized Controlled Trial
Source: JMIR Mhealth Uhealth. 2026 May 28;14:e71957. doi: 10.2196/71957 (PMC13218651; doi:10.2196/71957)
Supplement: Multimedia Appendix 1 [file mhealth-v14-e71957-s001.pdf]

Multimedia Appendix 1. Joint Effort mean scores on the user version of the Mobile App Rating Scale (uMARS) (Stoyanov et al., 2016), 2 weeks after baseline (N = 16/39)

Subscale/item		Mean (*SD*)[a]
Engagement		**3.84 (0.56)**
1	Entertainment	3.62 (0.89)
2	Interest	3.94 (0.57)
3	Customization	3.38 (0.62)
4	Interactivity	3.81 (0.83)
5	Target group	4.44 (1.03)
Functionality		**4.28 (0.52)**
6	Performance	4.19 (1.05)
7	Ease of use	4.25 (0.77)
8	Navigation	4.19 (0.91)
9	Gestural design	4.5 (0.63)
Aesthetics		**4.35 (0.55)**
10	Layout	4.5 (0.73)
11	Graphics	4.38 (0.72)
12	Visual appeal	4.19 (0.66)
Information		**4.34 (0.54)**
13	Quality of information	4.06 (0.77)
14	Quantity of information	4.62 (0.62)
15	Visual information	4.12 (1.26)
16	Credibility of source	4.56 (0.63)
Subjective items		**3.28 (0.62)**
17	Would you recommend	3.94 (1)
18	How many times	3.38 (0.89)
19	Would you pay	1.94 (0.77)
20	Overall (star) rating	3.88 (0.5)

[a]All items are rated on a 5-point scale from (1) “Inadequate” to (5) “Excellent.”
[b]User version of the Mobile App Rating Scale.
