# Supplementary material for: Acceptability, Feasibility, and Outcome Responsiveness of the Joint Effort Mobile App for Promoting Lower-Risk Cannabis Use Among Young Adults: Pilot Randomized Controlled Trial
Source: JMIR Mhealth Uhealth. 2026 May 28;14:e71957. doi: 10.2196/71957 (PMC13218651; doi:10.2196/71957)
Supplement: Checklist 1 [file mhealth-v14-e71957-s002.pdf]

**CONSORT 2010 extension for Pilot and Feasibility Trials Checklist (Eldridge, Chan, et al., 2016)**

| Section/Topic | Item No | Checklist item | Reported on page No |
|---|---|---|---|
| **Title and abstract** | | | |
| | 1a | Identification as a pilot or feasibility randomised trial in the title | Abstract |
| | 1b | Structured summary of pilot trial design, methods, results, and conclusions (for specific guidance see CONSORT abstract extension for pilot trials) | Abstract |
| **Introduction** | | | |
| Background and objectives | 2a | Scientific background and explanation of rationale for future definitive trial, and reasons for randomised pilot trial | Section 1 |
| | 2b | Specific objectives or research questions for pilot trial | Section 1.2 |
| **Methods** | | | |
| Trial design | 3a | Description of pilot trial design (such as parallel, factorial) including allocation ratio | Section 2.1 |
| | 3b | Important changes to methods after pilot trial commencement (such as eligibility criteria), with reasons | N/A |
| Participants | 4a | Eligibility criteria for participants | Section 2.2 |
| | 4b | Settings and locations where the data were collected | Section 2.2 |
| | 4c | How participants were identified and consented | Section 2.2 and 2.5 |
| Interventions | 5 | The interventions for each group with sufficient details to allow replication, including how and when they were actually administered | Section 2.3.2 |
| Outcomes | 6a | Completely defined prespecified assessments or measurements to address each pilot trial objective specified in 2b, including how and when they were assessed | Section 2.4 |
| | 6b | Any changes to pilot trial assessments or measurements after the pilot trial commenced, with reasons | N/A |
| | 6c | If applicable, prespecified criteria used to judge whether, or how, to proceed with future definitive trial | N/A |
| Sample size | 7a | Rationale for numbers in the pilot trial | Section 2.2 |

| | | | |
|---|---|---|---|
| | 7b | When applicable, explanation of any interim analyses and stopping guidelines | N/A |
| Randomisation: | | | |
| Sequence generation | 8a | Method used to generate the random allocation sequence | Section 2.3.2.3 |
| | 8b | Type of randomisation(s); details of any restriction (such as blocking and block size) | Section 2.3.2.3 |
| Allocation concealment mechanism | 9 | Mechanism used to implement the random allocation sequence (such as sequentially numbered containers), describing any steps taken to conceal the sequence until interventions were assigned | Section 2.3.2.3 |
| Implementation | 10 | Who generated the random allocation sequence, who enrolled participants, and who assigned participants to interventions | Section 2.3.2.3 |
| Blinding | 11a | If done, who was blinded after assignment to interventions (for example, participants, care providers, those assessing outcomes) and how | Section 2.3.2.3 |
| | 11b | If relevant, description of the similarity of interventions | N/A |
| Statistical methods | 12 | Methods used to address each pilot trial objective whether qualitative or quantitative | Section 2.6 |
| **Results** | | | |
| Participant flow (a diagram is strongly recommended) | 13a | For each group, the numbers of participants who were approached and/or assessed for eligibility, randomly assigned, received intended treatment, and were assessed for each objective | Figure 2 |
| | 13b | For each group, losses and exclusions after randomisation, together with reasons | Figure 2 |
| Recruitment | 14a | Dates defining the periods of recruitment and follow-up | Section 3.1.1 |
| | 14b | Why the pilot trial ended or was stopped | Section 2.4.2.1 |
| Baseline data | 15 | A table showing baseline demographic and clinical characteristics for each group | Table 1 |
| Numbers analysed | 16 | For each objective, number of participants (denominator) included in each analysis. If relevant, these numbers should be by randomised group | Table 3 |
| Outcomes and estimation | 17 | For each objective, results including expressions of uncertainty (such as 95% confidence interval) for any estimates. If relevant, these results should be by randomised group | N/A |
| Ancillary analyses | 18 | Results of any other analyses performed that could be used to inform the future definitive trial | N/A |

| | | | |
|---|---|---|---|
| Harms | 19 | All important harms or unintended effects in each group (for specific guidance see CONSORT for harms) | N/A |
| | 19a | If relevant, other important unintended consequences | N/A |
| **Discussion** | | | |
| Limitations | 20 | Pilot trial limitations, addressing sources of potential bias and remaining uncertainty about feasibility | Section 4.2 |
| Generalisability | 21 | Generalisability (applicability) of pilot trial methods and findings to future definitive trial and other studies | Section 4.2 |
| Interpretation | 22 | Interpretation consistent with pilot trial objectives and findings, balancing potential benefits and harms, and considering other relevant evidence | Section 4.1 |
| | 22a | Implications for progression from pilot to future definitive trial, including any proposed amendments | Section 4.3 |
| **Other information** | | | |
| Registration | 23 | Registration number for pilot trial and name of trial registry | Section 2.1 |
| Protocol | 24 | Where the pilot trial protocol can be accessed, if available | N/A |
| Funding | 25 | Sources of funding and other support (such as supply of drugs), role of funders | Funding section |
| | 26 | Ethical approval or approval by research review committee, confirmed with reference number | Section 2.7 |
